# Combined agonists act synergistically to increase mucociliary clearance in a cystic fibrosis airway model

**DOI:** 10.1038/s41598-021-98122-5

**Published:** 2021-09-22

**Authors:** Nam Soo Joo, Hyung-Ju Cho, Meagan Shinbashi, Jae Young Choi, Carlos E. Milla, John F. Engelhardt, Jeffrey J. Wine

**Affiliations:** 1 The Cystic Fibrosis Research Laboratory, Stanford University, Bldg. 420, Main Quad, Stanford, CA 94305-2130 USA; 2 Department of Pediatrics, Stanford University, Stanford, CA 94305 USA; 3 Department of Otorhinolaryngology, Yonsei University, Seoul, Korea; 4 Department of Anatomy and Cell Biology, Carver College of Medicine, University of Iowa, Iowa City, IA 52242 USA

**Keywords:** Physiology, Diseases, Medical research

## Abstract

Mucus clearance, a primary innate defense mechanism of airways, is defective in patients with cystic fibrosis (CF) and CF animals. In previous work, the combination of a low dose of the cholinergic agonist, carbachol with forskolin or a β adrenergic agonist, isoproterenol synergistically increased mucociliary clearance velocity (MCCV) in ferret tracheas. Importantly, the present study shows that synergistic MCCV can also be produced in CF ferrets, with increases ~ 55% of WT. Synergistic MCCV was also produced in pigs. The combined agonists increased MCCV by increasing surface fluid via multiple mechanisms: increased fluid secretion from submucosal glands, increased anion secretion across surface epithelia and decreased Na^+^ absorption. To avoid bronchoconstriction, the cAMP agonist was applied 30 min before carbachol. This approach to increasing mucus clearance warrants testing for safety and efficacy in humans as a potential therapeutic for muco-obstructive diseases.

## Introduction

Cystic fibrosis (CF) is a multi-organ syndrome of which the most critical clinical phenotype is the propensity for airway mucus obstruction, chronic lung infections, and neutrophilic inflammation. Unless arrested, the resulting tissue damage produces a life-long decline in pulmonary function. CF is caused by loss of function mutations in the gene for an anion channel, CFTR, cystic fibrosis transmembrane conductance regulator, which is important for fluid secretion in the airways. CF airways appear normal at birth, but their airway surface liquid (ASL) is less able to kill bacteria^1^ and mucus clearance rates are slowed^2^. Chronic lung infections are the main drivers of declining lung function in humans^3,4^, and when prevented in CF ferrets with prophylactic antibiotics, a tenfold increase in longevity ensued, in spite of still having a muco-obstructive phenotype with obstruction and inflammation^5^.

Improving mucociliary clearance velocity (MCCV) in CF airways is a therapeutic goal. Improvements in mucus clearance, sometimes sufficient to show clinical efficacy, have been obtained with inhalation therapies with recombinant human DNase (Pulmozyme)^6,7^, hypertonic saline^8,9^, or powdered mannitol^10^. For most people with CF, the most effective improvements in mucus clearance are provided by small molecules that partially restore CFTR function^11,12^. For those whose mutations are not treatable with current modulators, or whose lung declines continue despite modulators, additional improvements in mucus clearance could be therapeutic. MCCV is a function of the volume and rheological properties of airway surface liquid (ASL) and ciliary beat frequency (CBF)^13^. We define ASL as all the liquid on the surface, including both the periciliary layer, where shorter mucins are attached to the cilia^14^ and the mucus layer containing large gel-forming mucins like MUC5B, which are essential for mucus clearance^15^.

During experiments to quantify how CFTR and ENaC (the epithelial sodium channel) modulators alter mucociliary clearance in ex vivo ferret tracheas, we discovered that combinations of a β-adrenergic agonist (we used forskolin as a surrogate, but isoproterenol is effective) with a low dose of cholinergic agonist promoted dramatic increases in MCCV that were significantly greater than the sum of the increases produced by either agonist separately^16^. It was not known if these results would generalize, nor if they could be produced in CF.

Here, we show that such synergistic increases in MCCV can also be produced in pigs and importantly, in CF ferrets. We investigate the potential mechanism that leads to synergistic MCCV by studying the roles of airway submucosal glands, airway surface epithelia, and cilia.

## Results

### Synergistic increases of MCCV in CF ferrets and WT pigs

We define the ‘synergy paradigm’ as the sequential exposure to 30 min of either a cAMP or Ca^2+^ elevating agonist, followed by at least a 30 min exposure to the combined agonists. We used 10 µM forskolin, isoproterenol or formoterol as cAMP agonists, and 0.3 µM carbachol as the Ca^2+^ elevating agonist (all basolateral). In prior work these combinations caused increases in MCCV of ferret tracheas were much greater than the predicted additive effects of the two agonists and approached maximal values^16^.

**Figure 1:**
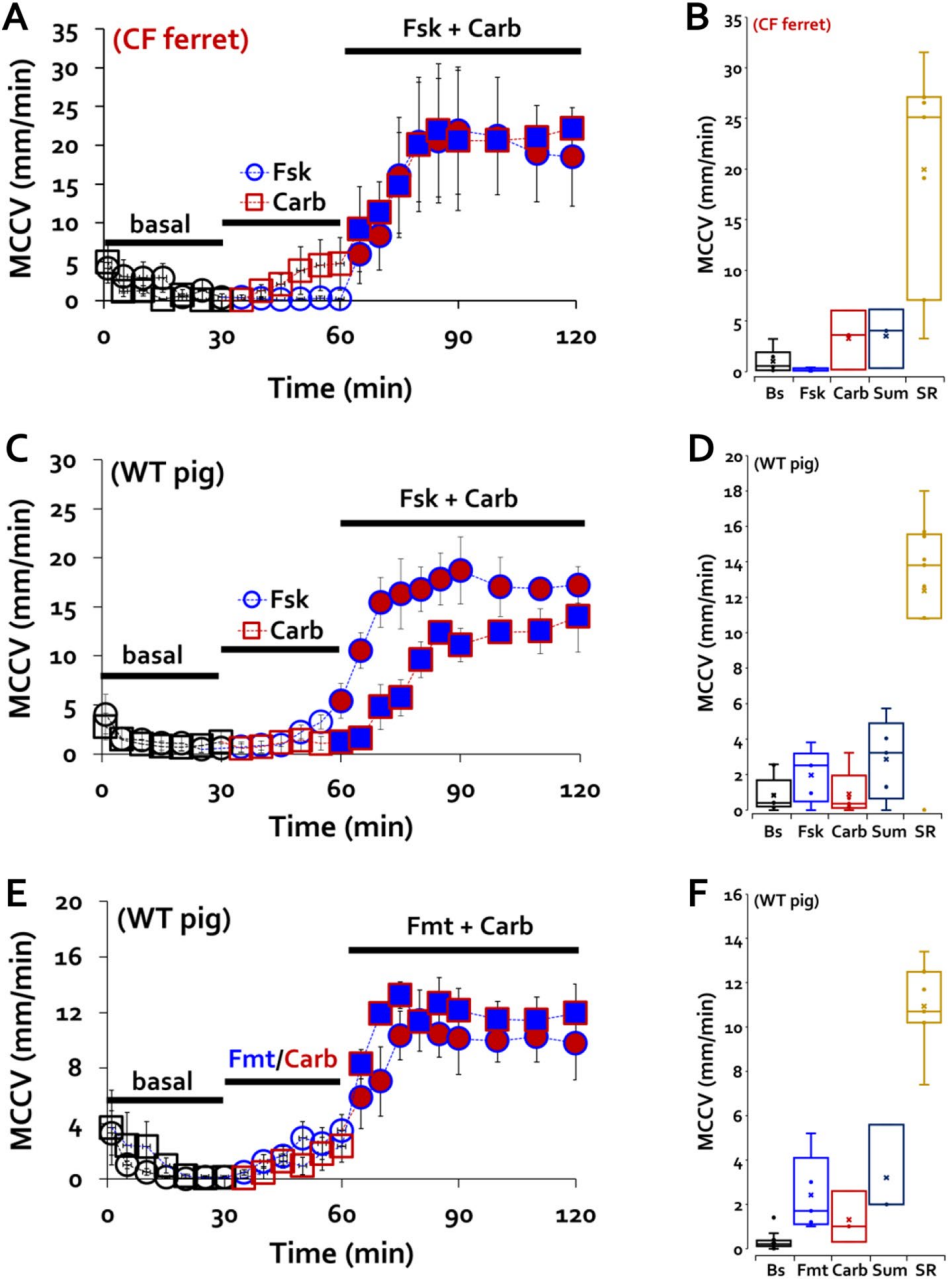
Synergistic mucus clearance in CF ferret and WT pig tracheas. (**A**) Time courses of MCCV from CF ferrets in response to 10 µM forskolin (blue open circles, Fsk, n = 4), 0.3 µM carbachol (red open squares, Carb, n = 4) or the combination (filled symbols). CF ferret genotypes were 5 CFTR^*G551D*^, one CFTR^*∆F/∆F*^, and one CFTR^*G551D/KO*^. (**B**) Summary data as box and whisker plots. Bs: basal/unstimulated MCCV, Sum: arithmetic sum of MCCV to agonists used separately, SR: synergy to combined agonists. SR was 5.7 times larger than sum, (*P* = 0.006, n = 3–7) (**C**) MCCV from 2 to 5-day old piglets: same protocol and symbols as for ferrets. (**D**) Summary data. SR was 3.9 times larger than sum, (*P* = 3.8E−05, n = 4–8). **(E)** Responses in pigs to 10 µM formoterol/Fmt (blue circles) instead of forskolin, otherwise same protocol and symbols. (**F**) Summary data. SR was 3.4 times larger than sum, (*P* = 0.005, n = 3–7).

To determine if synergistic increases of MCCV could be produced in CF ferret tracheas, we tested tracheas from 7 transgenic adult CF ferrets of mixed genotype (see “Methods” section). We divided the 7 CF ferret tracheas into two groups and tested 4 by applying forskolin first and 3 by applying carbachol first, followed by the combined agonists. MCCV (all values are in mm/min) was then measured for 90–150 min and plotted as MCCV vs time and agonist(s) in Fig. 1A. MCCV declined to near zero in the first 30 min without stimulation (Fig. 1A, see figure legend for details). Forskolin produced no increase in MCCV and carbachol produced only a small increase. However, when the agonists were then combined in either order, they produced large, sustained increases in MCCV to ~ 20 mm/min. Averaged data for the last 20 min of each period of basal and single drug treatment and the period from 10 to 80 min after adding the combined agonists are shown as a box and whisker plot in Fig. 1B. MCCV values were: unstimulated: 1.6 ± 1.09 (n = 3); by 10 µM forskolin: 0.18 ± 0.09 (n = 4); by 0.3 µM carbachol: 3.29 ± 2.08 (n = 3), ‘Sum’ is the arithmetic sum of MCCV induced by the two agonists used separately: 3.5 ± 2.07; SR, synergy response by the combined agonists: 19.95 ± 4.12, (*P* = 0.006 SR vs. Sum). Responses for individual CF ferrets are shown in Supplementary Fig. 1A–G.

Encouraged by these results, we then asked if synergy could be observed in different species. We measured MCCV in tracheas from 2 to 5 days old WT piglets (see “Methods” section for details). Unstimulated MCC velocity was less than 1 mm/min (averaged T10-30 MCCV, 0.93 ± 0.39, n = 8 piglet tracheas) like that of WT ferrets^16^. We divided the 8 piglet tracheas into two groups and tested 4 by applying forskolin first and 4 by applying carbachol first, followed by the combined agonists. Carbachol or forskolin alone produced only small increases in MCCV, but the combined agonists produced large, sustained increases in MCCV to 12–17 mm/min regardless of the order of addition (Fig. 1C). Averaged data for the last 20 min of each period of basal and single agonist treatment and the last 50 min of synergy paradigm period are shown as a box and whisker plot in Fig. 1D. Averaged MCCV values were: by 0.3 µM carbachol: 0.91 ± 0.63 to 1.12 ± 0.82 (*P* = 0.32, Carb vs. Bs/basal, 4 piglets); by 10 µM forskolin: 0.95 ± 0.65 to 2.46 ± 0.68 (*P* = 0.13, n = 4); sum of individual response (Sum): 3.58 ± 1.06, and by the synergy paradigm (SR): 13.92 ± 0.94.

The box and whisker plots make it clear that the increases in MCCV to the combined agonists were significantly larger for the combined agonists than for the arithmetic sum of the responses for both CF ferrets and WT pigs. Thus, MCCV synergy exists in at least two species and persists, at least in part, after loss of CFTR function. The MCCV of CF ferrets in the synergy condition was 5.7-fold faster than the arithmetically summed responses of agonists used separately, but the value of practical importance is how this compares to WT ferrets. We compared the CF ferret values with those obtained previously from WT ferrets^16^. For forskolin alone, WT versus CF ferret MCCV values were 6.75 ± 0.84 (n = 28) versus 0.18 ± 0.09 (n = 4). For carbachol alone WT versus CF ferret MCCV values were 8.24 ± 0.82 (n = 12) versus 3.29 ± 2.08 (n = 3). For synergistic responses to the combined agonists, WT vs CF MCCV values were 36.24 ± 0.9, (n = 40) versus 19.95 ± 4.12 (n = 7). Thus, compared to WT ferrets, CF ferret responses were ~ 0% for forskolin, ~ 40% for carbachol, and ~ 55% for synergy.

These experiments used forskolin to elevate cAMP. To evaluate a clinically readily available β-adrenergic drug, we measured MCCV in response to 10 µM of the β2-adrenergic receptor agonist formoterol in place of forskolin. We saw comparable synergistic increases in MCCV in pig tracheas (in mm/min): baseline, 0.3 ± 0.1 (n = 12); 10 µM formoterol, 2.4 ± 0.9 (n = 5), 0.3 µM carbachol, 1.3 ± 0.8 (n = 3); and 10.9 ± 0.8 by the combined agonists (n = 7 piglet tracheas) (Fig. 1E,F).

### Combined agonists did not induce airway smooth muscle contraction or airway narrowing

**Figure 2:**
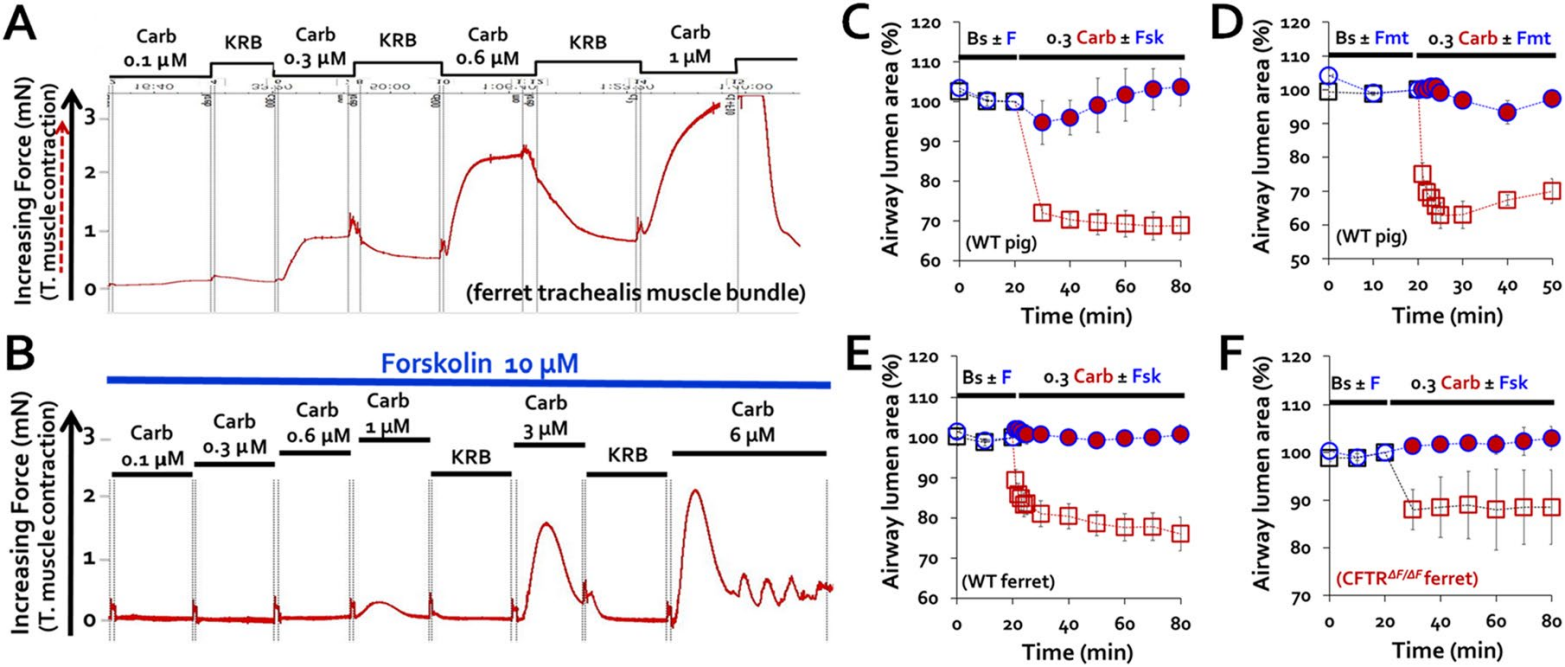
Elevating cAMP inhibited muscle tension and airway narrowing to carbachol. (**A**,**B**) To measure muscle tension, one end of an isolated WT ferret trachealis muscle bundle was secured in a Sylgard-lined Petri dish filled with KRB solution and the other end was attached by 26-gauge wire to a previously calibrated strain gauge. Tension responses to increasing carbachol doses in the absence (**A**) and presence (**B**) of 10 µM forskolin are displayed. (**C–F**) To measure airway narrowing, thin slices (~ 2 mm) of tracheal rings from WT pigs and WT or CF ferrets were treated with carbachol alone or in the presence of forskolin (F or Fsk) or formoterol (Fmt); their lumens were imaged over time and the areas measured as an assay for muscle constriction. After a baseline period (black open squares), carbachol was added (0.3 µM, red open squares), or in the presence of 10 µM forskolin or formoterol. (red closed circles). Averaged responses at 10 min intervals are shown for: (**C**) WT piglet tracheas (n = 3–7) with forskolin. (**D**) WT piglet tracheas with formoterol (n = 5). (**E**) WT ferret tracheas (2 tracheas, 5 experiments). (**F**). CF ferrets (n = 2, one CFTR^*∆F/∆F*^ and one CFTR^*∆F/G551D*^)**.**

The cAMP and Ca^2+^ -elevating agonists that increase MCCV also affect airway smooth muscle. Used alone they have opposite effects: Ca^2+^-elevating agonists contract muscles whereas cAMP-elevating agonists relax them. For therapeutic use, the potential for producing unwanted bronchoconstriction with the combined agonists is a safety concern. To determine which effect predominates, we measured airway smooth muscle responses to carbachol ± 10 µM forskolin or formoterol using two different methods: measuring muscle tension and lumen area. Tension of ferret trachealis muscle bundles was measured to increasing carbachol concentrations ± 10 µM forskolin. Forskolin abolished tension increases to 0.3 and 0.6 µM carbachol and greatly reduced responses to higher doses of carbachol (Fig. 2A,B). Lumen area, imaged in thin sliced piglet or ferret tracheal rings, displayed a sustained 20–40% reduction with exposure to 0.3 µM carbachol, but when carbachol was preceded by either forskolin or formoterol, it induced only a transient decrease of 5% or less (Fig. 2C–F). Importantly, this same protective effect was observed in CF ferrets (Fig. 2F).

The velocity of mucus clearance reflects the transportability of mucus and ciliary beat frequency. Transportability is in turn largely determined by hydration/concentration^17^ and pH (or bicarbonate content) of the mucus^18,19^. A major source of upper airway fluid is submucosal glands, and the agonists we used to stimulate MCCV also stimulate submucosal gland secretion^20–29^. ASL depth and composition are also modified by surface epithelia that secrete and absorb electrolytes/fluid. Indeed, this is the principle means of controlling ASL in airways that lack submucosal glands^30^. In prior work by us and others^26,31–33^ evidence was found for cholinergic inhibition of Na^+^ absorption, which would tend to increase the fluidity and transportability of mucus. Finally, CBF is increased by elevating either Ca^2+^^34^ or cAMP^35^. The following experiments sought evidence to support or challenge a possible contribution of each of these mechanisms to synergistic increases in MCCV.

### Synergistic glandular mucus secretion in WT pigs, WT ferrets, and CF ferrets

We hypothesize that synergistic increases in MCCV rely partly on increased mucus secretion from submucosal glands. This hypothesis arises from evidence that combinations of [Ca^2+^]_i_-elevating and [cAMP]_i_-elevating agonists produce synergistically elevated rates of mucus from submucosal glands of humans^22^, pigs^24^, and ferrets^21^. However, in those experiments different specific concentrations of agonists were used. To determine if the same protocols used here produced synergistic increases in secretion from submucosal glands, we measured mucus secretion rates of individual tracheal glands in WT pigs and ferrets and in CF ferrets via time-lapse optical imaging^28^ while stimulating them with the same concentrations of agonists and durations of exposure used for MCCV studies. All secretion rates are reported as nanoliters/min/gland.

**Figure 3:**
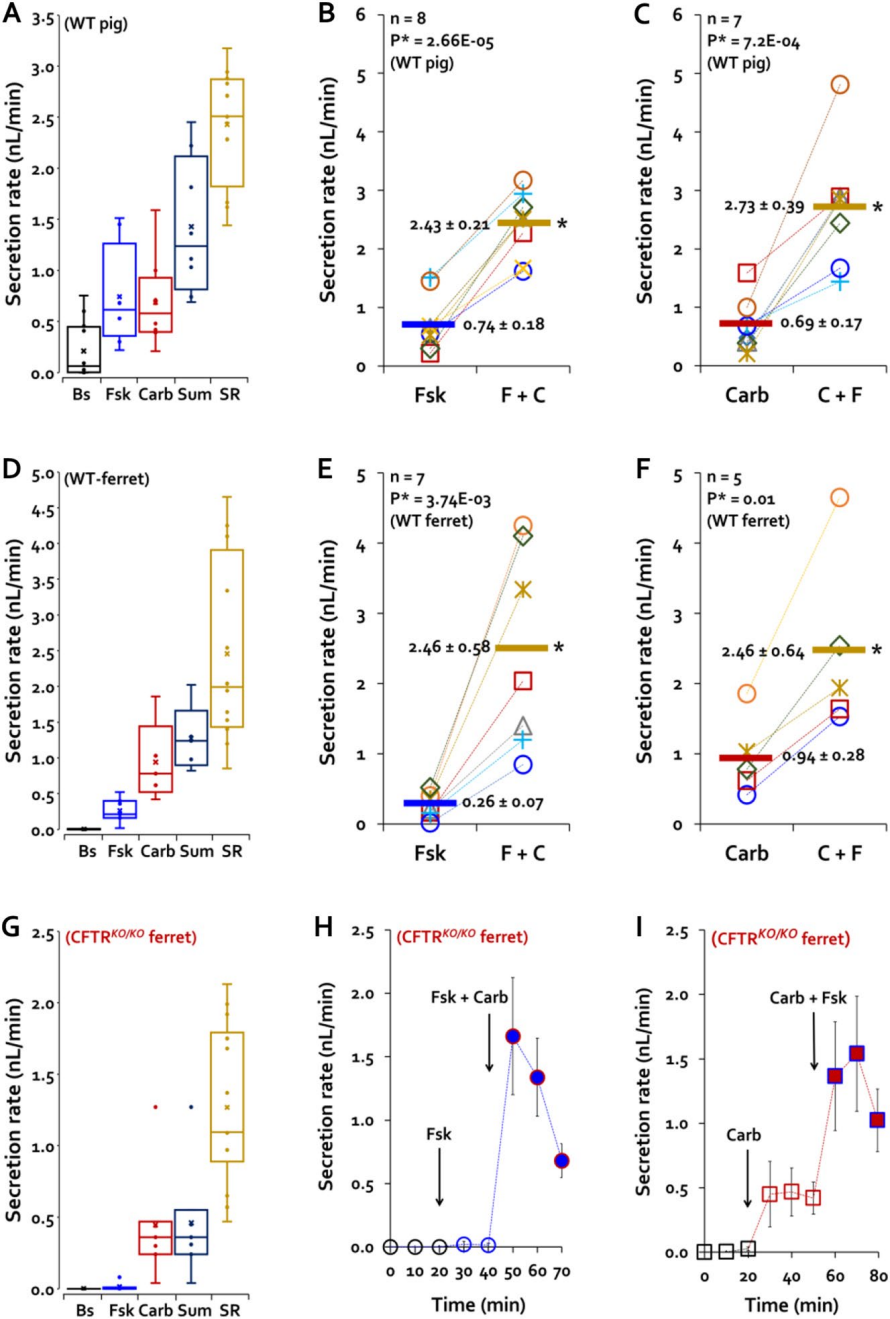
Combined agonists increase gland mucus secretion synergistically. Average secretion rates are summarized as box and whisker plots for 15 WT pigs (**A**), 12 WT ferrets (**D**) and 2 CF ferrets (**G**) using the same labeling as in Fig. 1. Each agonist increased secretion over baseline values, and rates to the combined agonists (SR) were significantly larger than the arithmetical sum (Sum) of their individual responses. (**B**) Average secretion rates for individual WT pigs to 10 µM forskolin alone and combined with 0.3 µM carbachol (60 glands, 8 pigs). (**C**) As in B but with carbachol alone and combined with forskolin (60–61 glands, 7 pigs). (**E,F**) WT adult ferret data with same conditions as in pigs (26–29 glands, 7 ferrets). (**H,I**) CF ferret data produced with same conditions as pigs and WT ferrets. A single ferret was run in each condition and secretion rates were measured in 7–14 glands. Time courses of average responses are plotted at 10 min intervals.

In WT pig tracheal glands, the average unstimulated secretion rate was in nl/min/gland, (0.21 ± 0.06, 121 glands, 8 pigs, Fig. 3A). The basal rate was significantly increased by each agonist alone and was further increased by their combination in either order. Rates to the combined agonists were significantly larger than the arithmetic sum of their individual responses: additive sum = 1.26 ± 0.19, 7 pigs versus combined agonists = 2.86 ± 0.25 (2.3-fold larger, *P* < 0.01, 8 pigs). Data for individual pigs is shown in Fig. 3B for forskolin first and in Fig. 3C for carbachol first. (See also Supplementary Movie 1).

WT Ferrets gave similar results (Fig. 3D–F). In ferret tracheal glands, the average unstimulated secretion rate was ~ zero (0.003 ± 0.001, 67 glands, 7 ferrets, Fig. 3D). It was significantly increased by forskolin (0.26 ± 0.07, 37 glands, 7 ferrets *P* < 0.05) and by carbachol (0.94 ± 0.28, 30 glands, 7 ferrets *P* < 0.05). The secretion rates to the combined agonists were significantly larger than the arithmetical sum of their individual responses (Fig. 3D): overall arithmetic sum = 1.27 ± 0.23 versus 2.46 ± 0.39 for the combined agonists (1.9-fold larger, *P* < 0.05, 55–67 glands, 5–7 ferrets. Data for individual WT control ferrets is shown in Fig. 3E for forskolin first and in Fig. 3F for carbachol first.

Importantly, CF ferrets (CFTR^*KO/KO*^) also showed synergistic gland secretion in spite of having no response to forskolin alone. We were able to test only two CF ferrets (Fig. 3G–I). One CF ferret was stimulated first with 10 µM forskolin, the other with 0.3 µM carbachol, and both with the synergy paradigm. Unstimulated secretion rates were ~ zero, as in WT ferrets. Forskolin alone failed to stimulate secretion as expected (0.01, 7 glands), carbachol alone increased the average secretion rate to 0.45 ± 0.16, and the combined agonists increased average rates to 1.23 ± 0.35, 7 glands, and 1.31 ± 0.19, 7 glands. When agonists were combined synergy was seen in both orders of addition. The averaged secretion rate across both ferrets to the combined agonists was 1.27 ± 0.15, which is 2.8 times the arithmetic sum of the two agonists used alone, and about half of the response of WT ferrets of 2.46 ± 0.39^16^.

To summarize this section, the rates of mucus secretion across both species and including CF ferrets is increased to values beyond the additive sum of the agents used alone, providing circumstantial evidence that gland secretion rates contribute to MCCV in our system.

### Combined agonists stimulate epithelial surface anion secretion and inhibit Na^+^ absorption

**Figure 4:**
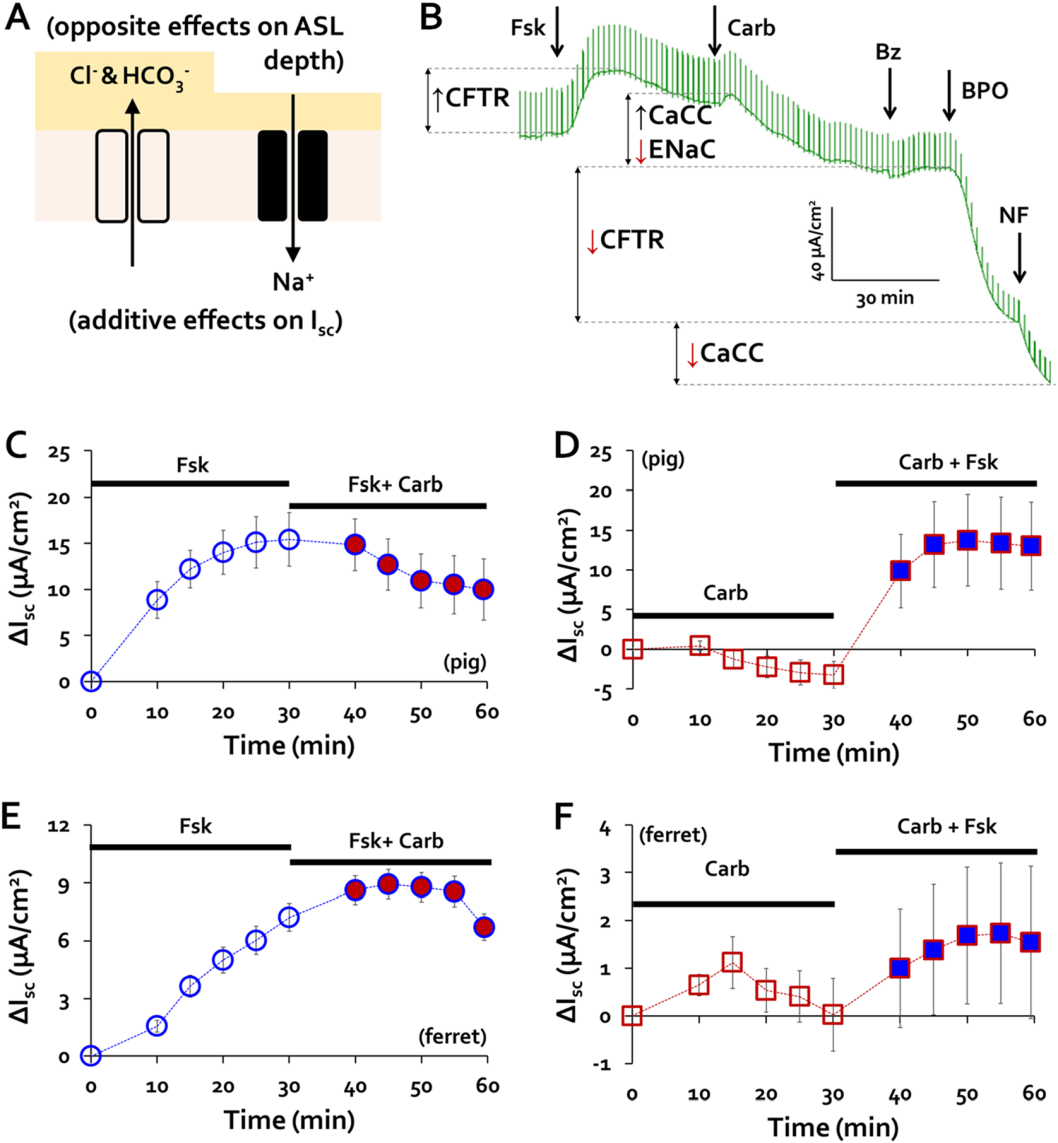
Combined agonists inhibit sodium absorption and stimulate anion secretion by surface epithelia. (**A**) Cartoon of two electrogenic, ion transport pathways across airway apical epithelium: anion secretion increases surface fluid, Na^+^ absorption decreases surface fluid. The two pathways have opposite effects on fluid height, but additive effects on I_sc_ because of their opposite valence and transport directions. (**B**) Raw trace of I_sc_ across pig tracheal epithelium using *Chart 4* software. After reaching a stable, unstimulated I_sc_ (here, > 2 h post-mounting), 10 µM forskolin, 0.3 µM carbachol, 10 µM benzamil, 20 µM benzopyrimido-pyrrolo-oxazinedione (BPO-27) and 200 µM niflumic acid were sequentially added at times shown (see text). (**C,D**) Pig tracheal mucosa: averaged ΔI_sc_ plots over time in response to (**C**) forskolin alone followed by forskolin + carbachol and (**D**) reversed order of agonist addition. (**E** & **F**) Ferret tracheal mucosa: averaged ΔI_sc_ plots with same protocol as for pigs. Pig traces (**C,D**) are based on 10–12 experiments with tissues from 6 to 7 pigs. Ferret traces (**E,F**) are from 7 experiments with tissues from 5 ferrets.

The surface epithelia also modify ASL. Figure 4A is a cartoon of the main ion flows controlling ASL depth: anion-mediated fluid secretion increases, and Na^+^-mediated fluid absorption decreases ASL depth. We hypothesize that the combined agonists increase ASL depth and thus MCCV by stimulating secretion and inhibiting absorption (see also Fig. 6). Figure 4B shows our best example of an I_sc_ trace from pig tracheal mucosa stimulated with forskolin followed by carbachol. Forskolin caused a sustained I_sc_ increase with no measurable change in conductance. When 0.3 µM carbachol was then added, it induced a transient I_sc_ increase followed by slow decreases in I_sc_ and conductance, with conductance reduced to 84% of the pre- and immediate post-forskolin value after ~ 30 min. The ENaC inhibitor benzamil (Bz) did not cause further changes in I_sc_ or conductance, suggesting that carbachol completely inhibited ENaC-dependent Na^+^ absorption. At this point the epithelium is secreting anions, indicated by steep drops in I_sc_ and conductance produced by the two anion channel inhibitors, BPO-27 and niflumic acid, with no counterbalancing absorption, so ASL depth is predicted to increase (dotted gold line in Fig. 6A) unless MCCV increases. Our evidence shows that MCCV does increase.

Figure 4C–F shows summary plots of ΔI_sc_ as a function of time and stimulation. Each panel shows responses to 10 µM forskolin or 0.3 µM carbachol for the first 30 min and then the combined agonists for the next 30 min for WT pigs (Fig. 4C,D**)** and WT ferrets (Fig. 4E,F). Forskolin increased ∆I_sc_ as expected for both species, but when carbachol was added the ∆I_sc_ diminished slowly (Fig. 4C,E). Our interpretation of I_sc_ in the forskolin + carbachol condition is that forskolin mainly increased I_sc_ by stimulating anion secretion while carbachol largely decreased I_sc_ by inhibiting Na^+^ absorption. Inhibiting Na^+^ absorption would increase net fluid accumulation on the surface. When carbachol is added first, the ΔI_sc_ decreased directly or after a transient increase (Fig. 4D,F). In both cases the subsequent ∆I_sc_ increase to carbachol + forskolin is smaller than to forskolin alone, because of their opposite effects on I_sc_ but additive effects on ASL depth.

### Agonist stimulation of human ciliary beat frequency (CBF)

**Figure 5:**
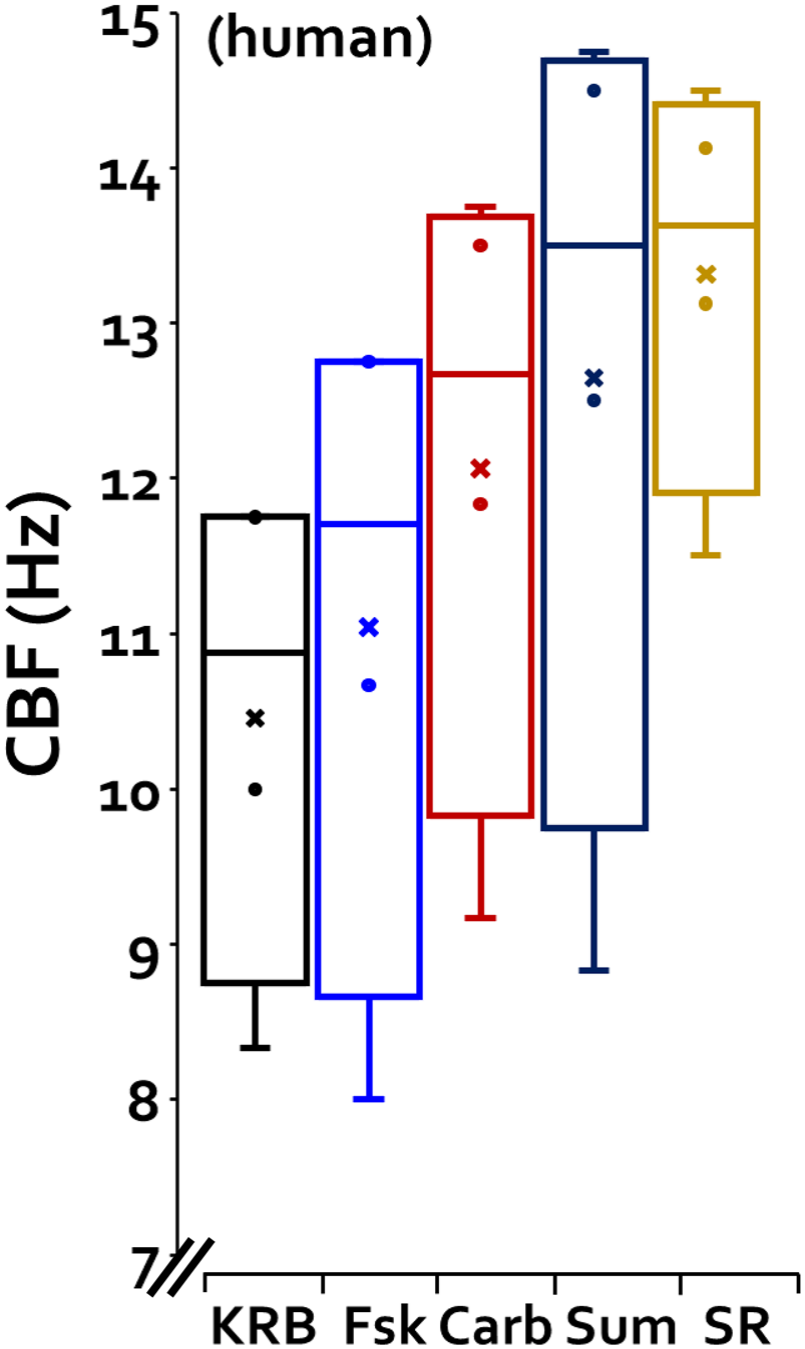
Agonists stimulated ciliary beat frequency with additive effects. Ciliary beat frequency was measured in human nasal mucosa from 4 patients. CBF (in Hz) of unstimulated tissues at 37 °C in Krebs buffer (KRB) was 10.46 ± 0.95. Agonists alone increased CBF by small amounts that did not reach significance in this small sample: carbachol: 11.04 ± 1.3 (5.3%) and forskolin 12.06 ± 1.22 (9.8%). The combined agonists increased CBF significantly 13.31 ± 0.77 (27.2%, n = 4, *P* < 0.05) when comparing difference in CBF (∆) to unstimulated CBF (KRB), but not to the arithmetical sum of ∆CBF to the combined agonists: 2.85 ± 0.76 (synergy paradigm) versus 2.19 ± 0.66 (arithmetic sum) (n = 4, *P* = 0.47).

CBF is known to increase in response to elevations of either [Ca^2+^]_i_^34^ or [cAMP]_i_^35^. We tested to see if CBF might display synergistic increases to the combined agonists. CBF (in Hz) of unstimulated human nasal cells in KRB (Krebs buffer solution) was 6.79 ± 1.69 at 25 °C and 10.46 ± 0.95 at 37 °C (4 subjects, *P* = 0.01) (see “Methods” section). As shown in Fig. 5, neither agonist increased CBF significantly, but when combined, their additive effects produced a 27.2% increase to 13.31 ± 0.77 Hz. This was a significant increase compared to unstimulated CBF (n = 4, *P* < 0.05), but not to the arithmetic sum of ∆CBF to the two agonists: combined agonists: 2.85 ± 0.76, and arithmetic sum: 2.19 ± 0.66 (n = 4, *P* = 0.47). Thus, while increases in CBF will contribute to increases in MCCV, they are unlikely to account for the synergistic increase in MCCV seen with the combined agonists (see “Discussion” section).

## Discussion

### Main findings

We have six main findings. (1) Combined agonists produced synergistic increase of MCCV in CF ferrets to 19.95 ± 4.12 mm/min, which is ~ 55% of MCCV in WT ferrets tested in similar conditions. (2) Little or no airway narrowing was produced by the combined agonists, even in CF ferrets. (3) Pigs also showed synergistic increases in MCCV, so the effect is not species-specific. As for mechanisms, we found: (4) synergistic increases in glandular mucus secretion in pigs, ferrets, and CF ferrets; (5) increased anion secretion and decreased Na^+^ absorption by surface epithelia; and (6) increased CBF, but only with an additive effect. The magnitude of synergistic MCCV increases were multiple-fold higher than to either agonist alone or to their summed responses and were close to maximal values reported in vivo. In anesthetized ferrets, basal MCCV in vivo was 18.2 ± 1.0 mm/min, and was increased to 32.0 ± 3.8 mm/min with maximal anticholinesterase treatment^36^. In anesthetized pigs, averaged basal MCCV in vivo was ~ 7 mm/min, and averaged maximal MCCV was ~ 12 mm/min^37^. If synergy also occurs in vivo, it should help mobilize mucus in certain obstructive airway diseases.

The most significant result was the synergistic increase in MCCV of CF ferrets. This is intriguing because in CF ferret tracheas forskolin alone did not increase MCCV (Fig. 1A) or stimulate gland mucus secretion (Fig. 3H). Also, using a different synergy paradigm human submucosal gland secretion was lost in airways from subjects with CF^22^. Therefore, the combined agonists used in the present study must be activating a CFTR-independent anion secretion pathway that is refractory to forskolin alone (see below).

### Strategies to increase mucus clearance

Strategies to increase mucus clearance are mainstays of cystic fibrosis treatment but are only modestly effective^6,8–10^. Pulmozyme (recombinant human DNase), hypertonic saline, and mannitol all improve mucus clearance in CF, while inhalation of bicarbonate or tromethamine improved CF sputum rheology^38^.

Long before Pulmozyme or hypertonic saline treatments, numerous studies documented that β-adrenergic (cAMP) agonists increased MCC^13,39^. Indeed β-adrenergic agonists, considered as bronchodilators, are now used ubiquitously for treating obstructive diseases. However, the doses needed to stimulate increased MCC are higher than those that reliably produce bronchodilation^39^, and so it is not clear to what extent the doses presently used are increasing MCC. Unlike β-adrenergic agents, cholinergic (Ca^2+^) agents cause bronchial constriction, which is the basis for the methacholine challenge test^40^, although increased mucus transport in humans by cholinergic stimulation has been reported^41^. Cholinergic agents also stimulate mucus secretion, and it is widely held that mucus over-production contributes to muco-obstructive disease^42,43^. Thus, it is not surprising that no one has previously advocated a therapeutic use for inhaling an agent that stimulates mucus secretion and causes bronchoconstriction. Indeed, anti-cholinergic agents are used as treatments for COPD, with modest effectiveness apparently resulting primarily from increased bronchodilation^44^. Thus, our finding that a combination of forskolin (or a β-adrenergic, formoterol) and a low-dose cholinergic markedly increased MCCV was unexpected.

**Figure 6:**
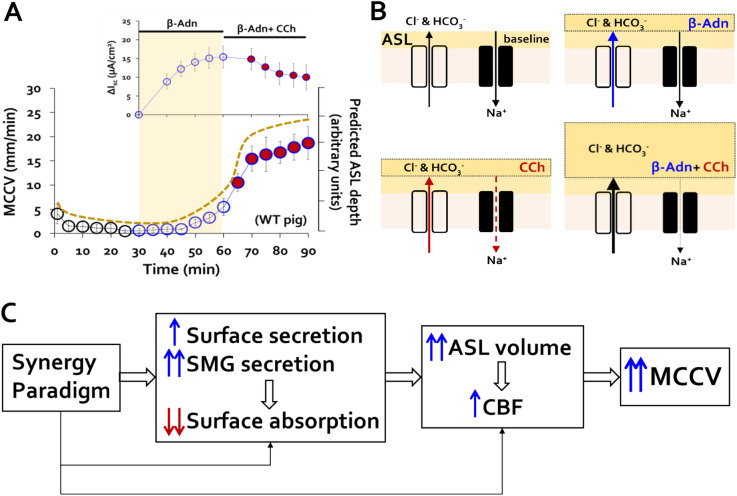
Summary diagram linking increased ASL to increased mucus clearance. (**A**) Relationship between ∆I_sc_, ASL depth and MCCV. MCCV (redrawn from Fig. 1C) is shown on the main graph; inset shows ∆I_sc_ (from Fig. 4C) with time points aligned to the MCCV graph. Dashed brown line is inferred change in ASL depth in the absence of MCC. (**B**) Cartoons of main electrogenic ion flows across tracheal surface epithelium in four conditions: baseline, β-adrenergic (β-Adn), carbachol (CCh), and β-Adn + CCh. Each panel shows the inferred status of anion secretion, Na^+^-absorption and resulting changes in ASL depth. The depth of ASL depth is inferred. Because carbachol inhibits Na^+^ (and fluid) absorption and stimulates anion (and fluid) secretion, the combined agonists have opposite effects on I_sc_, but at least additive increases ASL depth. (**C**) Summary diagram of component processes leading to synergistic increases in MCCV in ex vivo tracheas of WT ferrets, WT pigs and CF ferrets. The net result is a marked increase in MCCV.

Our hypothesis is that the combined agonists increase MCCV mainly because they increase ASL volume via three processes: synergistic increases in gland mucus secretion, increased fluid secretion and decreased absorption by surface epithelia (Fig. 6). The combined agonists produced only modest, additive increases in CBF measured in Krebs solution. It is possible that larger increases in CBF depend on increases in ASL volume, which occurred in the MCCV experiments but not in the CBF experiments. CBF increases have been observed using micro optical coherence tomography (μOCT) to visualize transport in intact tracheas^45,46^. Importantly, all of this occurs in the absence of airway narrowing.

The concept that MCCV will be faster if ASL depth is increased is supported by studies of patients with pseudohypoaldosteronism (PHA), where loss of function mutations in ENaC subunits eliminate Na^+^ absorption from the airway surface, which more than doubles the volume of ASL and causes a fourfold increase in 0–20 min clearance rates of inhaled tracer from the lungs^47^. Previously, we demonstrated that agonist-induced MCCV in ferrets was ~ doubled when ENaC was inhibited^16^. In those experiments^16^, stimulation with either forskolin or carbachol in the presence of ENaC inhibition increased MCCV to values similar to those seen with the combined agonists, providing additional evidence that synergistic MCCV results, in part, from ENaC inhibition. The idea that increased ASL provides faster clearance also underlies the logic of using β-agonists^39^ and hypertonic saline^8,9^ to increase clearance. It is also supported by studies of ex vivo pig tracheas, where stimulating secretion increased MCCV, blocking secretion slowed MCCV, and blocking absorption increased MCCV of tracheas after secretion had been blocked^20^.

### Potential molecular and cellular mechanisms

Molecular and cellular mechanisms responsible for synergistic MCCV by β-adrenergic and cholinergic agonists were not addressed in this study, but given our evidence that inhibition of ENaC contributes, prior works on molecular mechanisms of ENaC inhibition are relevant. A common theme is the role of elevated [Ca^2+^]_i_^48–50^, which can be achieved with a wide range of agonists, including ATP, UTP, histamine, thapsigargin, and bradykinin^51^. Cholinergic agonists increase [Ca^2+^]_i_; other mechanisms include increasing extracellular antiproteases^27,52^; and other ENaC inhibitors^25,53^ by stimulating secretions from airway glands and surface epithelia.

Because we observed synergistic increases of MCCV and glandular secretion in CF ferrets, mechanisms that bypass CFTR must be involved. Intracellular crosstalk between cAMP and Ca^2+^ signaling pathways via inositol 1,4,5-triphosphate receptor-binding protein release with IP_3_ (IRBIT) has been shown to mediate synergy in salivary gland and pancreatic ducts^54^. Synergistic secretion by lacrimal glands in response to cAMP and cholinergic agonists was partly due to inhibition of p44/p42 mitogen-activated protein kinase (MAPK) by the cAMP agonist^55^. A previous study^50^ demonstrated that synergistic fluid secretion by cAMP + Ca^2+^ agonists could result from Ca^2+^ release by a cAMP-dependent Ca^2+^ release mechanism in addition to a Ca^2+^ agonist in isolated serous cells from human nasal and WT & CFTR^-/-^ pig tracheal glands. Another study in HEK 293 cells^49^, however, has shown that a cAMP agonist, such as parathyroid hormone or isoproterenol, did not increase [Ca^2+^]_i_ by itself, but when combined with carbachol, a cAMP agonist potentiated carbachol-induced Ca^2+^ release by unmasking a discrete Ca^2+^ pool in ER. Discrepancies in previous reports might arise in part from using different cell or organ preparations and in part from using different measurement parameters, e.g., [Ca^2+^]_i_ versus [HCO_3_]_i_ ([pH]_i_). Our earlier studies^22,24^ have shown that there are CFTR-dependent and -independent paths in synergistic glandular mucus secretions, depending on the doses of β-adrenergic and cholinergic agonists.

### Potential therapeutic relevance for muco-obstructive disease

Procedures to enhance mucociliary clearance are needed for people with muco-obstructive airway disease, including substantial numbers of people with CF^11,56^. Because β-agonists and methacholine are used routinely (the latter to test for hyperactive airways), little should stand in the way of testing them in combination except that it seems counterintuitive. Our ex vivo data show this combination is effective in speeding mucus clearance without inducing airway narrowing, even in CF animals (Fig. 2), which have airway muscles with increased sensitivity to cholinergic agonists^57^. However, as we found with WT ferrets, the combined agonists minimizes/prevents airway narrowing induced by carbachol in WT pigs and in CF ferret airways. Our results are consistent with an earlier study where greatly reduced bronchoconstriction was observed when a β-adrenergic agonist was administered prior to methacholine in CF children^58^.

It remains to be seen if this combination is safe in individuals with hyperactive airways. If results do warrant further testing in people with CF, it will be important to start early with healthier airways, because the trend observed with β-agonist improvement of MCC was that healthier airways showed more benefit than diseased airways^39^.

## Materials and methods

### Airway tissue procurement

#### CF ferret tissues

Seven transgenic CF ferret tracheas (five CFTR^*G551D/G551D*^, one CFTR^*∆F508/∆F508*^, one CFTR^*G551D/KO*^) were used for MCC assays. These ferrets were raised on the CFTR modulator VX770. Dosing was stopped at least 3 weeks prior to euthanasia; no residual drug effect is expected or was observed (zero response to forskolin). Two CFTR^*KO/KO*^ ferret tracheas were used for the tracheal, single gland mucus secretion rate assay. Two CF ferret tracheas (one CFTR^*∆F508/∆F508*^ and one CFTR^*G551D/∆F508*^) ferret trachea were used for tracheal smooth muscle contraction assay. All isolated CF ferret tracheal trims (2–3 cm in length) were placed in DMEM culture medium immediately after euthanasia and shipped from the University of Iowa via overnight priority express.

#### Pig tissues

Newborn WT piglet tracheas (2–5 days old) were directly obtained at the swine facility of UC-Davis or from the laboratory of David Stoltz, University of Iowa, via overnight priority express. Postmortem (< 1 h) tracheas from young adult *Yorkshire* pigs (30–50 kg) and 5–12 months old *M. putorius* ferrets were from animal facilities at Stanford and Gilroy/CA. All methods using animal tracheae were carried out in accordance with relevant guidelines and regulations of Stanford University and animal protocols were approved (Stanford IACUC protocol#: 10,048). Piglet tracheas were shipped in DMEM cell culture medium, other animal tissues were transported to the laboratory in cold PhysioSol™ solution (Hospira, IL/USA) and then transferred to ice-cold Krebs Ringer bicarbonate (KRB) buffer gassed with 95% O_2_ and 5% CO_2_ and kept at 4 °C until use. The KRB buffer contained (in mM): 115 NaCl, 25 NaHCO3, 2.4 K2HPO4, 0.4 KH2PO4, 1.2 MgCl2, 1.2 CaCl2, 10 glucose, and 1.0 µM indomethacin, adjusted to pH 7.2 and ~ 290 mOsm at room temperature.

#### Human tissues

Human nasal mucosal tissues were obtained from nasal biopsies during endoscopic sinus surgeries at Yonsei University Hospital. All methods using human tissues were carried out in accordance with relevant guidelines and regulations of Yonsei University, Seoul, South Korea. All experimental protocols were approved by Yonsei University and informed consent was obtained from all participants prior to the study (Yonsei University-IRB protocol#: 4-2016-1153).

### MCCV measurement

Details are in the previous reports^16,59^. Briefly, each whole length ferret or piglet trachea, or a CF tracheal trim, was cut open along the mid-dorsal line and mounted mucosal side up onto a Sylgard elastomer platform. For pig experiments, we used tracheas from newborn piglets because tracheas from adult pigs that had been subjected to acute experiments, our source for submucosal gland experiment, had erratic MCC velocities due to epithelial damage secondary to intubation. The prepared trachea was placed into a sealed, humidified chamber bubbled continuously with gas (95%/5%-O_2_/CO_2_) with the serosal surface bathed with KRB buffer ± drugs. For the initial 30 min stabilization period, the tissue was submerged in the bath as the temperature was gradually increased to 37 °C. Then excess apical solution was drained, and tissue was incubated for an additional 10 min before starting baseline measurements of MCCV. Drugs were added by bath replacement with pre-warmed bath + drug(s). Summary MCCV data are reported as a single number and based on the averaged MCCV during the last 20 min of the treatment period unless stated otherwise.

### Electrophysiology

Intact ferret tracheal trims (~ 0.5 × 1.0 cm^2^) or pig mucosal preparations dissected from cartilage were mounted in EasyMount Ussing chambers (Physiologic Instruments, CA, USA) with exposed surface areas of 0.45 cm^2^, bathed in KRB buffer at 37 °C, and gassed with 95% O_2_/5% CO_2_. Transepithelial short-circuit current (I_sc_) was obtained and displayed with a VCC-600 voltage clamp (Physiologic Instruments, CA/USA), and a PowerLab *Chart4* software (V. 4.1.2, https://adinstruments.com, ADInstruments, CO/USA). Total tissue conductance was calculated by applying Ohm’s law to the I_sc_ deflection resulting from a 1 mV pulse across the tissues every 20 s during the experiment. We report averaged responses for the last 20 min of each measurement period unless stated otherwise.

### Optical measurement of glandular mucus secretion rate

Details are in the previous report^28^. Intact ferret tracheal trim (~ 1.5 cm^2^) or pig tracheal mucosa dissected from the underlying cartilage in cold Krebs Ringer bicarbonate buffer was mounted mucosal side up in a 35 mm Petri dish lined with pliable silicone so that the glands were bathed in KRB buffer while the surface was dried and covered with water-saturated mineral oil. The appearance of “mucus bubbles” (Supplementary Movie 1) within the oil layer was visualized by oblique illumination and digital images were captured with the macro lens of a Nikon digital camera. Stored images were analyzed by direct measurement or with *ImageJ* software (V. 1.50i, https://imagej.nih.gov/ij/, NIH, MD/USA). Rates for the indicated drugs were calculated for 5 min intervals based on averaging sustained T10-30 secretion rates by 10 µM forskolin or 0.3 µM carbachol alone or T5-30 by the combined agonists to include bubbles to be merged rapidly caused the combined agonists (see Supplementary Movie 1).

### Ciliary beat frequency measurement

Ciliary beat frequency was measured using human nasal mucosa in the lab where ferret and pig tracheal mucosa was not readily accessible. Human nasal mucosa from endoscopic nasal biopsies was further dissected under a microscope and placed in a chamber controlled for temperature and pH control. Perfused Krebs bicarbonate buffer was maintained at 37 °C and pH 7.4. Cilia were visualized with a Zeiss microscope equipped with 40 × or 60 × objectives (Munich, Germany) using differential interference contrast (DIC) optics. Images were viewed live and were captured automatically at 2,000 fps with a high frame-rate digital camera (optiMOS and NIS-Elements microscope imaging software (Nikon, Japan)) and converted to TIFF images. Images were obtained for 10 s at each condition and experiments were performed in the following sequence: (1) unstimulated CBF at room temperature; (2) unstimulated at 37 °C; (3) CBF at 37 °C with 0.3 μM carbachol; (4) wash for 10 min; (5) CBF with 10 μM forskolin; and (6) CBF with 0.3 µM carbachol added to the forskolin. Each condition was maintained for at least 10 min. Note that this paradigm differs from the synergy paradigms used for measuring MCCV and gland mucus secretion rates in that the exposure to agonists was ≥ 10 min instead of 30 min, and it omits the condition in which forskolin was added in addition to carbachol. All recordings at each condition were made at three different areas of the epithelia, and the analyzed CBF was averaged for each experiment. To analyze captured images and calculate CBF, an in-house coding with a *MATLAB* software (MA, USA) was used.

### Airway smooth muscle contraction measurement

Two methods were used to measure tracheal smooth muscle contraction. One is designed to measure airway narrowing using thin sliced tracheal rings. Piglet or ferret tracheal ring preparations of ~ 2 mm were submerged and securely pinned on a Sylgard-lined Petri dish filled with KRB solution at 37 °C and pH 7.4. Digital images of tracheal ring contractions in response to agonists for 1–10 min intervals were recorded with a Nikon digital camera and the inner lumen surface area of the tracheal ring was calculated using *ImageJ* (NIH, MD/USA). The other method is using a force transducer. One end of an isolated ferret trachealis muscle bundle was secured in a Sylgard-lined Petri dish filled with KRB solution and the other end was attached by 26-gauge wire to a previously calibrated strain gauge (series 400A force transducer system, Cambridge Technology, MA/USA). Tension responses to increasing carbachol doses ± 10 µM forskolin were obtained and displayed with PowerLab *Chart4* software (ADInstruments, CO/USA).

### Reagents

Chemicals were purchased from Sigma-Aldrich (St. Louis, MO/USA), Calbiochem (Billerica, MA/USA), and Alomone labs (Jerusalem, Israel). BPO-27 was a generous gift from Alan Verkman, UCSF. Forskolin, benzamil, BPO-27, niflumic acid, formoterol fumarate were dissolved in dimethyl sulfoxide (DMSO) and carbachol was dissolved in sterile double distilled water and indomethacin was dissolved in absolute ethanol. Solutions were made fresh or maintained at − 20 °C as aliquots of stock concentration. All chemicals were diluted 1:1000 with bath KRB solution (except indomethacin, 1: 10,000) immediately before use at the concentrations indicated.

### Statistics

Data is presented as mean ± S.E.M. unless otherwise indicated. To compare means of different treatment groups, we used either Student’s paired and unpaired *t*-test or *Mann–Whitney U* test.

## Supplementary Information

Supplementary Video 1.

Supplementary Information 1.
