# Supplementary material for: Combined agonists act synergistically to increase mucociliary clearance in a cystic fibrosis airway model
Source: Sci Rep. 2021 Sep 22;11:18828. doi: 10.1038/s41598-021-98122-5 (PMC8458446; doi:10.1038/s41598-021-98122-5)
Supplement: Supplementary file 2 — Supplementary Information 1. [file 41598_2021_98122_MOESM2_ESM.docx]

**SUPPLEMENTARY MATERIALS**

Original title: Synergistic mucociliary clearance–a novel approach to improve impaired mucociliary clearance in cystic fibrosis airways

(Revised title: Combined agonists act synergistically to increase mucociliary clearance in a cystic fibrosis airway model)

Nam Soo Joo, Hyung-Ju Cho, Meagan Shinbashi,

Jae Young Choi, Carlos E. Milla, John F. Engelhardt and Jeffrey J. Wine

**Supplementary Figure 1. Individual MCCV responses for 7 CF ferrets.**

**Supplementary Movie 1. Synergistic glandular mucus secretion by the combined agonists in pigs**.

**Supplementary Figure 1.** Individual MCCV responses for 7 CF ferrets to the synergy paradigm. (A-G) Protocols and genotypes are shown on each time-MCC velocity plot. Note that a reduced y-axis scale was used (E) and (G) to show synergistic MCCV. Also note that the averaged MCCV of T10-30 to 0.3 µM carbachol in (E) is less than 5% of those in (A) and (C).

**Supplementary Movie 1 (Movie-S1).**

Synergistic glandular mucus secretion by the synergy paradigm in pigs. The movie is composed of basal glandular secretion for 20 min, secretion by 0.3 µM carbachol for 30 min, and followed by synergy paradigm for additional 30 min with 10 min intervals in a pig tracheal mucosal preparation. The glandular mucous bubbles are shown at the area covered by water-saturated mineral oil.

Please open the movie file with VLC media player to properly view the movie.

To download VLC media player software — https://www.videolan.org/vlc/index.html
